# Transparent crosslinked ultrashort peptide hydrogel dressing with high shape-fidelity accelerates healing of full-thickness excision wounds

**DOI:** 10.1038/srep32670

**Published:** 2016-09-07

**Authors:** Wei Yang Seow, Giorgiana Salgado, E. Birgitte Lane, Charlotte A. E. Hauser

**Affiliations:** 1 Institute of Bioengineering and Nanotechnology, 31 Biopolis Way, Singapore 138669, Republic of Singapore; 2 Institute of Medical Biology, 8A Biomedical Grove, Singapore 138648, Republic of Singapore; 3 Laboratory for Nanomedicine, King Abdullah University of Science and Technology, Thuwal 23955-6900, Saudi Arabia

## Abstract

Wound healing is a major burden of healthcare systems worldwide and hydrogel dressings offer a moist environment conducive to healing. We describe cysteine-containing ultrashort peptides that self-assemble spontaneously into hydrogels. After disulfide crosslinking, the optically-transparent hydrogels became significantly stiffer and exhibited high shape fidelity. The peptide sequence (LIVAGKC or LK_6_C) was then chosen for evaluation on mice with full-thickness excision wounds. Crosslinked LK_6_C hydrogels are handled easily with forceps during surgical procedures and offer an improvement over our earlier study of a non-crosslinked peptide hydrogel for burn wounds. LK_6_C showed low allergenic potential and failed to provoke any sensitivity when administered to guinea pigs in the Magnusson-Kligman maximization test. When applied topically as a dressing, the medium-infused LK_6_C hydrogel accelerated re-epithelialization compared to controls. The peptide hydrogel is thus safe for topical application and promotes a superior rate and quality of wound healing.

The rapid healing of a wound is a fundamental health concern and has been studied for more than 2000 years^1^. Today, wound healing continues to burden healthcare systems worldwide. For instance, in the United States an estimated three to six million patients suffer from wounds that do not heal properly and this translates into a healthcare bill of more than $3 billion annually^2^.

Wound healing consists of four distinct but overlapping processes (hemostasis, inflammation, proliferation and remodeling) and there is a wealth of comprehensive reviews on this topic^1^,^3^,^4^. Each process is highly orchestrated and can directly impact the subsequent cascade of events. Briefly, in a healthy body, any incisional wound to the skin rapidly activates the formation of a fibrin clot to reduce bleeding and opportunistic infection. Neutrophils and monocytes, which can differentiate into macrophages, are among the first inflammatory cells to arrive on site to eliminate pathogens which have crossed the skin barrier^3^,^4^. This is followed by a phase of intense proliferation as fibroblasts (the main cell population of the dermis, the inner tissue of skin) upregulate their secretion of collagen (the bulk constituent of the dermis) and other extracellular matrix (ECM) components. Meanwhile, keratinocytes (the main cell population of the epidermis, the outer tissue layer) at the wound edges start to migrate towards the center to seal off the wound. New blood vessels begin to sprout to feed the frenzy of cellular activities. Some fibroblasts also differentiate into myofibroblasts^1^, which are believed to act by physically pulling the skin edges together in a centripetal fashion, leading to wound closure and contraction^5^. Excessive contraction, however, causes scarring which may compromise function and aesthetics. Finally, the dermal ECM is remodeled from being rich in collagen type III to one that is predominantly collagen type I^4^, while blood vessels and (myo)fibroblasts that are no longer required die by apoptosis^3^. The newly regenerated skin does not achieve its original tensile strength^5^, and depending on the depth of injury, appendages such as hair follicles and sweat glands may never reform^3^. Many factors including age, diet and psychological or disease state (diabetes is a potent antagonist) affect the rate of wound healing^2^.

Hydrogels^6^ have a useful water-swollen three-dimensional network. Herein lies the premise for using hydrogels in wound repair as they offer a moist environment for healing, which is more favorable for re-epithelialization than a dry one^7^,^8^. Various products are commercially available to aid the healing of cutaneous (as opposed to internal deep-tissue) wounds and the reader is referred to an excellent article for a detailed overview^9^. Many such products are obtained from hydrocolloids, alginate, polyurethane, silicone or nylon. They can also contain charcoal for odour management or antiseptics like iodine and silver^9^. However, these products have reached their developmental end-points and opportunities for further improvements are limited. Moreover, iodine and silver dressings are not recommended for chronic applications^9^. New materials are therefore currently under investigation for wound healing applications and many are being developed as hydrogels. Selected examples include hydrogels based on natural polymers such as chitosan^7^,^10^,^11^,^12^, dextran^13^, alginate/gelatin^8^ and collagen/glycosaminoglycan^14^. For instance, a bi-layer chitosan hydrogel was well tolerated and encouraged skin reconstruction by pigs with third-degree burns^11^. An amine-functionalized dextran hydrogel crosslinked with polyethylene glycol diacrylate promoted neovascularisation and regeneration of skin appendages in mice with burn wounds^13^. Alginate dialdehyde was used to crosslink gelatin in the presence of borax to form hydrogels and applied to rats with excision wounds^14^. Re-epithelialization, however, was only significantly different from the no-treatment control at the earlier, but not later time points.

On the other hand, hydrogels can be formed using synthetic polymers. For example, fibroblast-encapsulated hydrogels derived from poly(ethylene glycol)-poly(L-alanine) accelerated wound closure and re-epithelialization in rats with excision wounds compared to when gel or cells were absent^15^. In another study, a poly-L-lactic acid hydrogel grafted with RGD was used to encapsulate endothelial progenitor cells^16^. This formulation then promoted vascular regeneration in mice with excision wounds compared to when the cells were simply injected intradermally. Other formulations include tetracycline-loaded nanosheets fabricated from chitosan/sodium alginate/poly(vinyl acetate) which prolonged the survival rates of mice bearing bacteria-infected burn wounds compared to mice left untreated or treated with nanosheets without antibiotic^17^. Membranes made from silk were also loaded with epidermal growth factor and silver sulfadiazine and demonstrated to promote re-epithelialization in mice with excision wounds^18^. Concerns over the use of naturally-derived materials include their chemical heterogeneity and the risk of immunogenicity and pathogen transmission^19^,^20^. Conversely, synthetic materials are more defined chemically but tend to require significant efforts in synthesis or purification^21^. Furthermore, harmful or expensive reagents, solvents or catalysts may also be involved.

Our group focuses on peptide-derived hydrogels^22^,^23^. We have identified a series of amino acid (AA) sequences comprising three to seven residues that can assemble spontaneously in water to form hydrogels^24^,^25^. These ultrashort peptides obtained from naturally-occurring AA are chemically defined, biocompatible and can be easily bulk-produced using standard solid phase peptide synthesis techniques. Importantly, their short length confers a comparative cost advantage over other protein- or polypeptide-based hydrogels. Previously, we described another lysine-containing ultrashort peptide hydrogel that promoted the healing of partial-thickness burn wounds, albeit in reference to a commercially available dry wound dressing control^26^. In that study, we evaluated a physical gel whose fibers depended solely on physical forces for self assembly. Consequently, when soaked in a large volume excess of water, the fibers can get diluted, causing the gel to lose its shape^27^. We have since optimized the original sequences and introduced cysteine-mediated disulfide crosslinks into our rational peptide design^27^. As a result, the gels attained significantly greater stiffness, elasticity and shape fidelity even after a prolonged period of water immersion^27^. These modified gels can also be easily functionalized. For instance, the well-known integrin binding sequence, CRGD, was conjugated onto the peptide backbone via hydrogen peroxide (H_2_O_2_)-mediated disulfide bond formation^27^.

We now explore and further characterize this family of cysteine-containing peptides. We tested the allergenic potential of a selected peptide candidate with the Magnusson-Kligman maximization assay in order to evaluate its suitability for skin application. Previously, we reported on partial-thickness burn wounds^26^. Compared to an excision wound, the recovery characteristics of a burn wound are mostly different. For instance, the boundaries of a burn wound are less defined as the source of heat used to create the injury can exert effects greater than the area of contact. Also, in a burn model, the level of debridement of damaged tissue can vary at the start of experiment. Thus, the monitoring of recovery can be complicated. In an excision model, however, the precisely-created wound boundaries allow for easy surveillance. We therefore topically applied several peptide hydrogel formulations to mice with full-thickness excision wounds and investigated their wound healing properties with respect to a commercial hydrogel dressing.

## Results and Discussion

With LIVAGKC (LK_6_C), we have introduced a pioneer cysteine-containing peptide^27^. This sequence featured a hydrophobic tail with a string of AA arranged to give a gradient of hydrophobicity. This was followed by a hydrophilic headgroup capped with a cysteine for crosslinking. Disulfide bonds between cysteines were then formed by H_2_O_2_-mediated oxidation. Crosslinked gels were significantly stiffer than non-crosslinked counterparts and had better shape integrity even after water immersion, thus facilitating handling. Finally, excess H_2_O_2_ was quantitatively removed from the gel using a simple dialysis-inspired method prior to bioapplication^27^.

**Figure 1:**
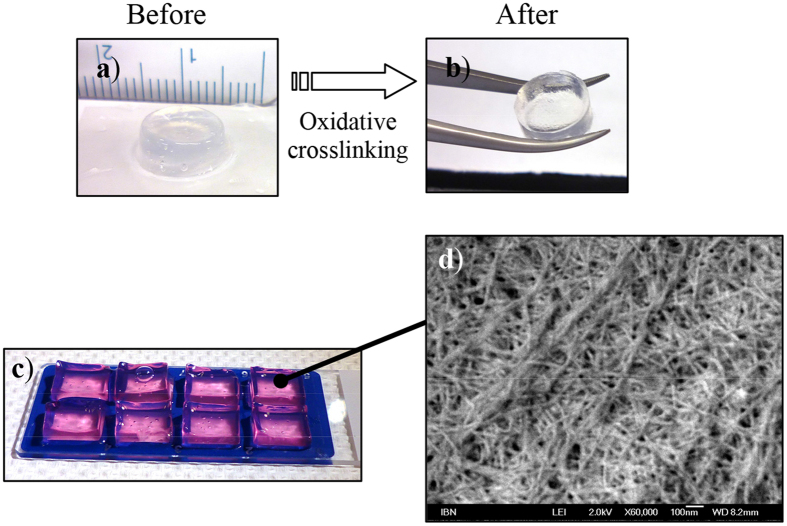
(**a,b**) The gel stiffness increased after crosslinking and can be easily picked up with a pair of forceps, while remaining optically transparent. (**c**) LK_6_C + CRGD gels (99% water) were clear, transparent and easily handled. Phenol red was added to aid visualisation. d) FESEM analysis revealed a dense mesh of fibrous network. The thickness of the fibers was on the order of tens of nm, mimicking the fibrous microenvironment found in the ECM.

A powerful advantage of peptide-based systems is that properties can be optimized at the single AA level, allowing for a great amount of control. Here, we investigate the relationship between sequence and physical property by making key mutations to the sequence of LK_6_C. As seen from Table 1, each sequence had its unique gelation concentration. The stiffness of the gels, as reflected by the elastic modulus (G′) value, generally increased after oxidative crosslinking. As a result, selected crosslinked gels can now be easily handled with forceps (Fig. 1a,b). This degree of physical manipulation is not possible prior to crosslinking and greatly facilitates their application during surgical interventions. However, removing the cationic K headgroup (LC_6_), or substituting it with an anionic D (LD_6_C) or neutral S (LS_6_C) residue interfered with the solubility and reproducibility to form gels. Switching the position of I and L in the hydrophobic tail (IK_6_C) resulted in gels that lost transparency, required a higher gelation concentration and became less stiff. There appears to be a correlation between stiffness and transparency, consistent with the suggestion that stiffer gels tended to be more transparent due to less phase separation or precipitation^28^. Shorter analogues (the tetramers) could also form gels with varying properties.

We proceeded to evaluate the peptide hydrogels in the context of cutaneous wound healing. From a clinical perspective, the ideal dressing allows monitoring of a healing wound *in situ* without having to remove the dressing^17^. This is not always possible with currently available dressing products, but may be possible with a clear transparent gel prepared from LK_6_C or IVKC. Despite being less stiff then IVKC gels, crosslinked LK_6_C gels could still be easily handled for applications in wound healing. Moreover, LK_6_C had a cost advantage over IVKC due to its lower gelation concentration. LK_6_C was therefore selected for further evaluation. Acetic acid as the counterion was also selected in preference to the trifluoroacetic acid formulation to achieve greater biocompatibility and to further reduce cost, as the acetic acid formulation required a lower gelation concentration.

Clear, transparent and easy to handle blocks of gel made from crosslinked LK_6_C (10 mg/mL) functionalized with CRGD (1 mg/mL) were cast in the format used for subsequent *in vivo* study (Fig. 1c) and analyzed by field emission scanning electron microscopy (FESEM). This revealed a dense mesh of fibrous network which enabled the gel to trap >99% water within its bulk (Fig. 1d). The fibers were generally tens of nm thick and fell within the diametric lengthscale (5–300 nm) of fibers found in the ECM^29^.

**Figure 2:**
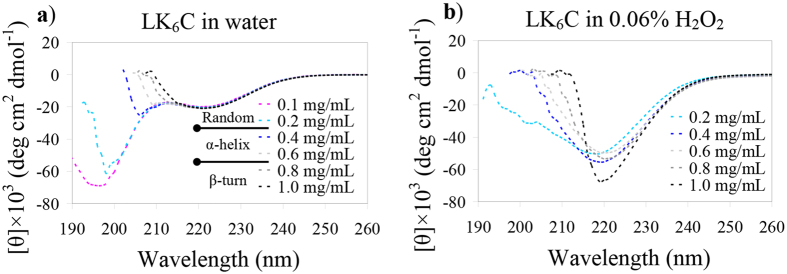
CD studies performed with LK_6_C either in (**a**) water or (**b**) 0.06% H_2_O_2_. (**a**) The secondary structure of LK_6_C transited from being predominantly β-turn to α-helical, followed by random coil as it was serially diluted in water. (**b**) In H_2_O_2_, LK_6_C made the transition from being predominantly β-turn to α-helical at a lower concentration of 0.2–0.4 mg/mL, compared to 0.6–0.8 mg/mL in water. This suggests that the disulfide bridges restrict the peptide fibers to a greater extent, thereby reducing their propensity to change secondary conformations.

We previously showed that crosslinked LK_6_C gels remained intact after a prolonged period of water immersion whereas non-crosslinked gels rapidly lost their bulk shape^27^. This high shape fidelity is presumably due to a fiber “lock in” effect by the intra- and inter-chain disulfide bonds. To confirm this, we conducted a series of circular dichroism (CD) experiments. LK_6_C gels (+/− H_2_O_2_) were first formed at 10 mg/mL and their CD signals recorded. Supplementary Fig. S1a shows that both gels possessed secondary structures rich in β-turns, as evidenced by the characteristic negative bands at ~200–225 nm^30^. However, upon serial dilution of LK_6_C gels formed in water (Fig. 2a), a gradual destabilization of the β-turn conformation was observed. This was reflected by a blue shift of the negative band^30^,^31^. Commensurate with this was an increasing α-helical content characterized by the double minima at ~208 and 222 nm^30^. A random coil conformation (negative peak at ~195–200 nm) eventually resulted, latest by 0.2 mg/mL. In comparison, the transitions between conformations were significantly delayed for crosslinked LK_6_C gels (Fig. 2b). For instance, the transition from β-turn to α-helix occurred at a lower concentration of 0.2–0.4 mg/mL, compared to 0.6–0.8 mg/mL in water. The gel also failed to adopt a random coil conformation even at 0.2 mg/mL. This suggests that disulfide-crosslinked fibers were more spatially constrained and less likely to alter their secondary structure. Interestingly, a survey of the depth of the minima at ~220 nm^32^ revealed that crosslinked LK_6_C gels exhibited the most pronounced β-turn character (Fig. S1b). Non-crosslinked gels or gels crosslinked in the presence of CRGD had reduced β-turn character.

To ascertain the allergenic or sensitization potential of the peptide before application to a wound, a Magnusson-Kligman maximization test was conducted. LK_6_C dissolved in water was administered to the skins of guinea pigs following a regime consisting of an induction phase by intradermal injection, a topical application phase and a subsequent challenge phase. Animals in the negative control group received a phosphate-buffered saline solution (PBS) while the positive control group received 0.1% dinitrochlorobenzene (DNCB) in 95% ethanol. Skin patches were then graded based on the severity of irritation according to the Magnusson and Kligman scale described in Table 2. As can be seen, all animals gained weight and showed no apparent signs of toxicity throughout the study. DNCB, however, provoked an allergic response in all animals within the positive control group, whereas both LK_6_C and PBS failed to elicit any obvious signs of sensitivity. This confirms that LK_6_C had no or, at worst, weak allergenic potential.

**Figure 3:**
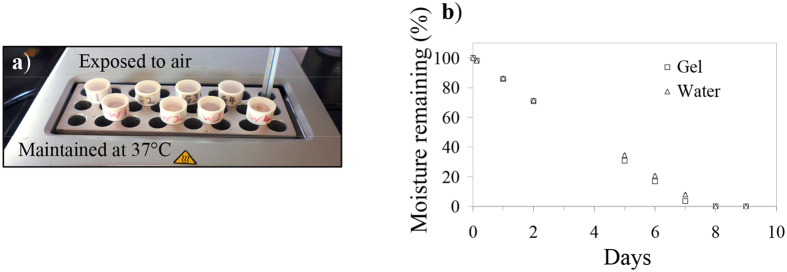
Rate of water lost from wounds covered with Tegaderm. (**a**) Small containers were filled with 200 μL of either water or LK_6_C + CRGD hydrogels and sealed with Tegaderm. The containers were then left on a heated plate (37 °C) exposed to ambient conditions to simulate the wound/air interface. (**b**) The weight of the containers was then recorded regularly to quantify the amount of moisture remaining and reported as average ± s.d. of quadruplicates. The rate of water lost was linearly constant and there was no significant difference between the samples.

The premise for using hydrogels in wound repair is that it offers a moist environment conducive to healing. We were thus interested to estimate the rate of water lost from the peptide gel across Tegaderm, which is a semi-permeable polyurethane adhesive membrane used in our subsequent *in vivo* wound healing study. Small containers were filled with water or LK_6_C + CRGD gel and sealed with Tegaderm. To simulate the wound/air interface, the containers were left on a heated plate maintained at 37 °C with the membrane-sealed top exposed to air (Fig. 3a). Figure 3b shows that the rate of water lost from the water or gel sample was linear and not significantly different. This indicates that while the peptide nanofibers efficiently entrapped water, they offered no additional barrier for evaporative water lost. Under current experimental conditions, it required close to seven days to dehydrate the peptide gels. This suggests that the gel can moisten the wound for at least seven days during which a change of dressing may not be needed, assuming the gel stays physically intact. In the context of an actual wound, however, the gel can be expected to stay hydrated even longer as the presence of wound exudates should reduce the rate of hydrogel dehydration.

We went on to perform *in vivo* wound healing experiments with mice. There are important differences in the skin anatomy and wound healing behaviour of rodents compared to humans^33^. For example, rodent skin is more elastic, loosely connected to the underlying tissue and pronounced contraction plays a significant role during wound closure. Human skin, conversely, is tighter and tends to heal mainly by re-epithelialization. Yet despite such caveats, testing on animals is still extremely informative, not least because of the ease of obtaining skin biopsies for analysis. Rodents are especially popular animal models due to their affordability and the ability to generate knock-out strains. It is therefore common practice to start with small rodents for skin research before progressing to larger animals such as pigs, which are much costlier but more similar to humans in terms of skin and wound healing characteristics^11^.

**Figure 4:**
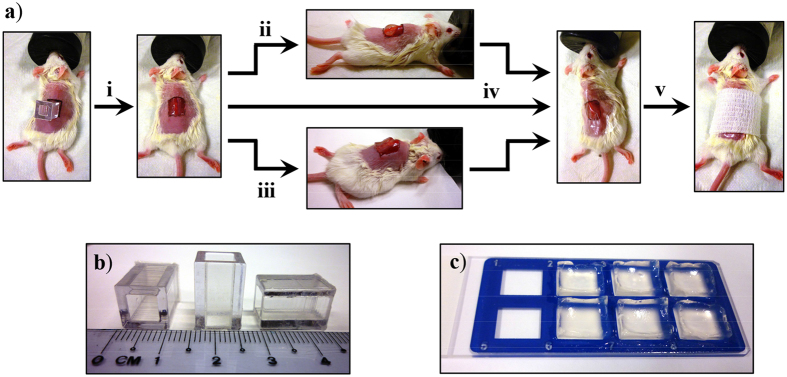
(**a**) Schematic of the full-thickness excision mouse model used to evaluate the wound healing property of LK_6_C + CRGD hydrogel dressings. (**i**) The wound boundaries were marked out on dorsally shaved mice using a template (as shown in **b**). Both the epidermis and dermis were then surgically removed to create 1 × 1 cm full-thickness wounds. Either the (**ii**) positive control (DuoDerm hydroactive gel) or (**iii**) peptide hydrogel (cast as ready-to-use sterile gel blocks, as shown in **c**) was applied to the wounds. (**iv**) All wounds were then covered up with Tegaderm, including the negative control group which received no gel application. (**v**) The wound was lightly bandaged and left to heal. Animals were sacrificed on day 3, 7 and 14 for histological examination.

A schematic of the surgical procedure is provided in Fig. 4. Full thickness excision wounds (1 × 1 cm) were created on all mice by surgically removing the epidermis and dermis. A block of the LK_6_C + CRGD gel or the DuoDerm hydrocolloid gel (positive control) was then applied topically onto the wound. All wounds, including the negative controls which received no gel, were finally covered with Tegaderm. DuoDerm was selected as a positive control because of its widespread clinical usage and comparability to the peptide hydrogel in terms of physical properties. Tegaderm was chosen because it allows gaseous and vapour exchange but, unlike traditional cotton gauzes, does not soak up moisture from the gels. Prior to application, residual H_2_O_2_ was removed from the peptide gel with a dialysis-inspired technique. To achieve isotonicity, the peptide gel was also infused with a common serum-containing tissue culture medium henceforth referred to as completed medium (see Experimental section). Usefully, the sodium pyruvate present in medium helped to remove any remaining traces of H_2_O_2_ which may cause toxicity^34^, while the provision of nutrients may potentiate wound healing.

**Figure 5:**
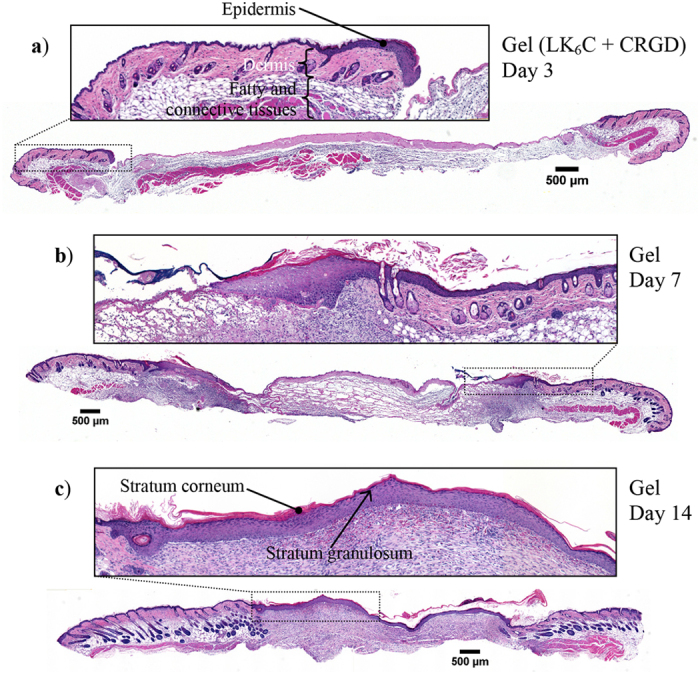
Representative cross-sectional histological sections of wounds treated with peptide hydrogel infused with completed medium. Skin samples were obtained on day (**a**) 3, (**b**) 7, (**c**) 14 for staining with H&E. Over time, the leading front of the epidermal “tongue” was observed to migrate inwards to close up the wound and restore the critical barrier function of skin. Re-epithelialization was completed by day 14, along with the appearance of a visible layer of stratum corneum and stratum granulosum. All insets show magnified images of the respective boxed regions.

Mice were sacrificed on day 3, 7, 14 and cross-sections of the wounds at maximum diameter were obtained for hematoxylin and eosin (H&E) staining. Figure 5 shows representative images of H&E stained wounds of mice treated with the peptide gel infused with completed medium. There was minimal re-epithelialization and deposition of granulation tissue at day 3 (Fig. 5a). Most of the wound was therefore still exposed. The epidermis at the wound edge was visibly hyperproliferative, in contrast to unwounded skin where the murine epidermis is typically two to four cell layers thick. By day 7, re-epithelialization was underway and the leading edge of the epidermis, the epidermal “tongue”, could be clearly observed (see inset, Fig. 5b). Granulation tissue had formed and was denser at the wound edge compared to the middle. By day 14, re-epithelialization was complete and a thick layer of granulation tissue was observed (Fig. 5c). Importantly, a prominent layer of stratum corneum and stratum granulosum, accounting for the critical barrier function of skin, had formed. The newly regenerated epidermis was still several cell layers thick and lacked appendages.

**Figure 6:**
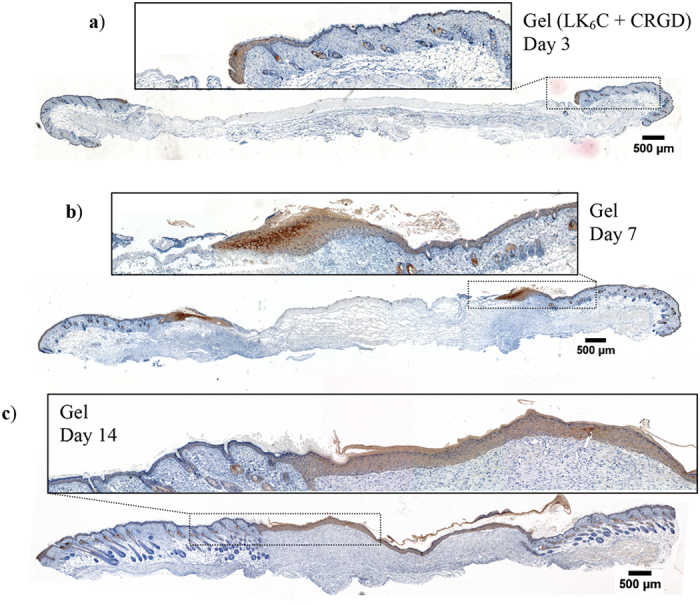
Representative cross-sections of wounds stained for K14 protein (using monoclonal antibody LL001). Wounds were treated with the completed medium-infused peptide hydrogel and animals were sacrificed on (**a–c**) day 3, 7 and 14 for immunohistochemistry. K14 is a marker for basal keratinocytes, as evidenced by its basal localization in unwounded epidermis. In these healing wounds, K14 was detected in both the basal and suprabasal layers of the newly regenerated epidermis. This suggests that keratinocytes in the newly regenerated epidermis have yet to fully differentiate as the neo-epidermis gradually matures to a more homeostatic morphology (2–4 cell layers thick).

To aid visualization of the epidermal “tongue”, staining for the keratin 14 (K14) protein was performed (Fig. 6). As expected, K14 immunohistochemical staining was localized to the basal layer in unwounded epidermis and hair follicles. In the wounded skin, however, K14 expression was detected in both the basal and suprabasal layers of the newly regenerated epidermis. This is indicative of a still immature neo-epidermis, as expected at this stage. All wounds in the different treatment groups exhibited a similar K14 staining pattern.

**Figure 7:**
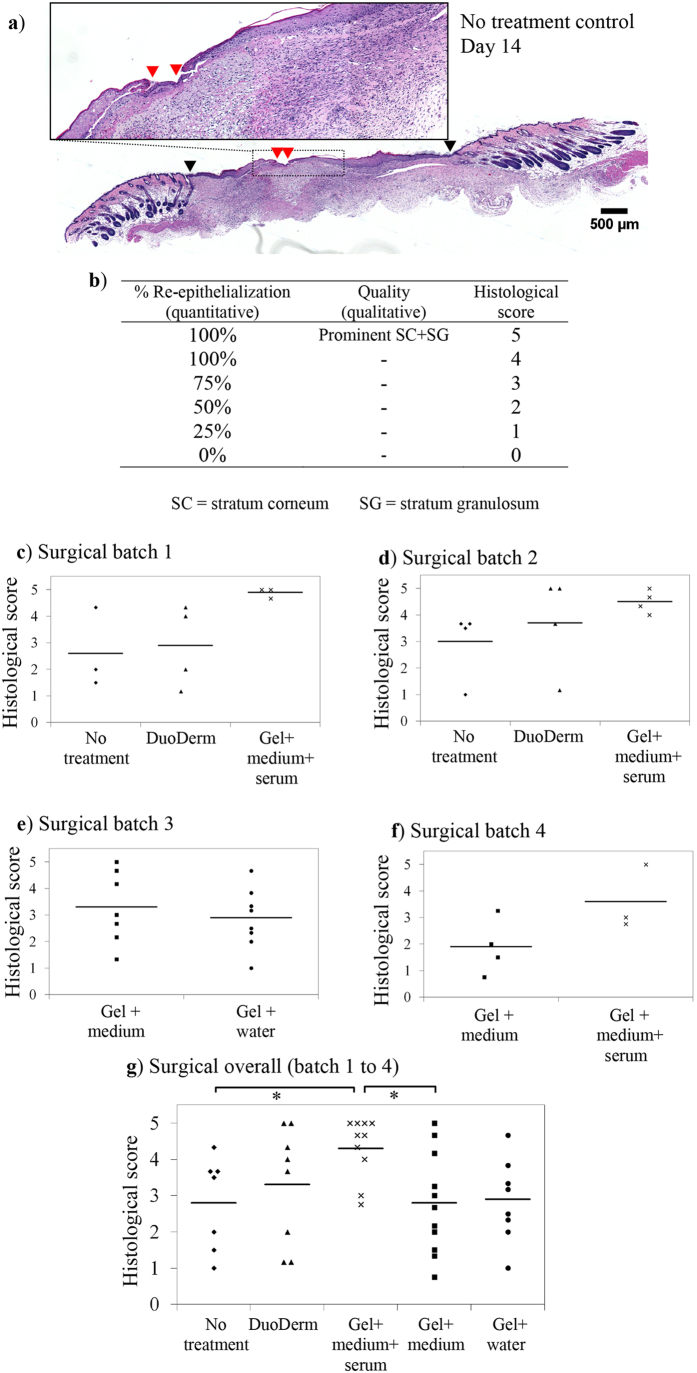
Comparison of different treatment strategies on day 14. (**a**) Representative H&E cross-section of a wound from the no-treatment group. The black arrow heads mark out the edges of the original excision sites, upon which the wound gap remaining (red arrow heads) was normalized against to derive the % re-epithelialization achieved. (**b**) The scoring criteria used in this semi-quantitative scale for grading skin samples. (**c–f**) Surgery was performed over four batches of animals. Skin sections were obtained on day 14 and scored based on the quality of re-epithelialization. (**g**) The overall scores of all treatment groups (n = 7–11). Wounds treated with peptide hydrogel infused with completed medium (average group score: 4.3) received higher and more consistent histological scores than wounds treated with the DuoDerm gel positive control (average: 3.3), no-treatment negative control (average: 2.8), or peptide gels infused with medium alone (average: 2.8) or water (average: 2.9).

To discriminate between different treatment groups, H&E stained tissues were obtained on day 14 for scoring (n = 7–11). A critical feature of the skin is its barrier capability. Therefore, a scale based mainly on the % re-epithelialization was devised. For illustration, a skin section from the no-treatment group is shown in Fig. 7a where the wound gap remaining was identified by red arrow heads. Next, the edges of the original excision sites (black arrow heads) were identified for normalization to derive the % re-epithelialization achieved. This was then converted into a histological score where a maximum score of five was awarded to wounds healed with a continuous epidermis and a prominent layer of stratum corneum and granulosum (Fig. 7b). The scores of three independent observers were averaged and reported for each tissue. Surgery was performed over four batches of animals (Fig. 7c–f). As can be seen from the overall plot in Fig. 7g, the completed medium-infused peptide hydrogel-treated group (average group score: 4.3) fared significantly better (p < 0.05) and received more consistent scores compared to the no-treatment negative control group (average: 2.8). This group also achieved a marginal improvement (p = 0.09) over the DuoDerm-treated positive control group (average: 3.3). Peptide gels infused with medium alone (average: 2.8) or water (average: 2.9), however, performed worse than peptide gels infused with completed medium.

**Figure 8:**
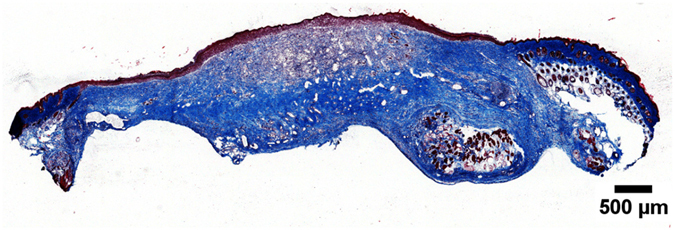
Representative frozen cross-section of a wound treated with the completed medium-infused peptide hydrogel after Masson’s trichrome staining on day 14. Collagen is stained blue. Collagen deposition was denser near the wound edge and in the deeper tissue layers, indicative of the inwards and upwards progression of wound healing. There was no obvious difference between the treatment groups in terms of density and location of collagen deposition.

Masson’s trichrome staining was used to investigate the density and location of collagen expression (collagen stained blue, nuclei black and muscle/cytoplasm/keratin red) on day 14. Figure 8 reveals that collagen, as expected, was mainly deposited in the dermis and granulation tissue. A density gradient was also observed where collagen deposition was generally denser near the wound edges and in the deeper layers of the skin. This reflects the chronology of collagen synthesis and wound repair – i.e., healing occurs inwards and upwards. The wound center healed last and therefore, had less collagen deposition. It was difficult to construct an objective scale to grade the extent of collagen deposition. Nonetheless, there are no visually obvious differences among the treatment groups in terms of the density and location of collagen deposition (Fig. S2).

A primary outcome of cutaneous wound healing is to regain a continuous layer of cornified epidermis so as to restore the barrier capability of skin. Scarless healing, which occurs during embryonic wound repair for instance^3^, represents the optimal aesthetic outcome of wound healing. One study suggested that a shorter healing time reduced the risk of hypertrophic scar formation^35^. A caveat, however, is that data was obtained with paediatric burn patients, which may not be totally translatable to adults. Nonetheless, rapid wound healing is a desirable outcome. Our results suggest that accelerated wound healing may be promoted by the topical application of peptide hydrogel infused with completed medium. The medium can be incorporated either during or after gel formation. In the latter, a plain gel can be soaked within the selected medium.

The presence of serum and hence, growth factors, was shown to be important. In an experiment, we attempted to use serum-infused medium without gel as a treatment group, i.e, dripping the medium over the wound before Tegaderm application (data not shown). However, a limiting technical difficulty of this procedure was that the medium flowed uncontrollably, resulting in widely-varying doses being applied to each animal. A hydrogel formulation which could immobilize the medium was therefore necessary for consistent application. Currently, such a hydrogel formulation is not readily available in the market. We now show that this strategy can potentially guide the design of next-generation dressings to treat wounds with higher efficacy than regular gel dressings. In the clinical context, a potential source of serum or growth factors may be derived from the patient undergoing the procedure. This also avoids concerns over immunogenicity and the cost associated with recombinant growth factors.

In neither the peptide gel nor DuoDerm treated groups was the gel bulk still clearly visible by day 7. Similar disappearance of gel material has also been reported by other studies^12^,^36^. Presumably, the fibrous constituents of the gel scaffold (composed of helical fibers^24^,^25^) could have been absorbed by the tissue or degraded by proteolysis. It can also be physically disintegrated during animal movement or be dislodged under the loose murine skin. Thus the effects of gel application may be more relevant to the earlier cascade of events, which then could have activated positive knock-on effects on the subsequent wound healing processes. Nonetheless, our data clearly showed that the peptide gel dressing, when infused with completed medium, improved the rate and quality of wound healing even with only a single dose application (i.e., no re-application) of gel to the animals.

## Conclusion

In this study we generated and analyzed a library of cysteine-containing peptide hydrogels. CD experiments suggest that disulfide bond formation kept the fibers (tens of nm in diameter) together and accounted for the high shape fidelity of crosslinked gels even after prolonged periods of water immersion. One sequence (LK_6_C) was then chosen for *in vivo* evaluation on mice with full-thickness excision wounds. Crosslinked LK_6_C gels are handled easily with forceps during surgical manipulation and offer an improvement over our earlier study of a non-crosslinked peptide hydrogel for burn wounds. LK_6_C had limited allergenic potential as it failed to provoke any sensitivity when administered to guinea pigs in the Magnusson-Kligman maximization test. More importantly, when applied as a topical wound dressing, the completed medium-infused LK_6_C hydrogel potentiated re-epithelialization compared to controls. This peptide hydrogel formulation was thus demonstrated to be safe for topical application and facilitated a superior rate and quality of wound healing. Since the formulation of the peptide gel was demonstrated to be important, LK_6_C gels can, in future, be infused with other media or active ingredients to further improve efficacy.

## Experimental

### Casting of peptide hydrogels and rheology

Peptides were synthesized by American Peptide Company (CA, USA) with >95% purity, N-terminus acetylation and a net peptide content of 70–90%. The gross weight of the peptide powder was used for all calculations in this study.

Gels were cast in hollow rings with diameters of 10 mm. Peptide powder was dissolved directly in either water or PBS to the preferred working concentration (see Table 1). If oxidation was desired, 0.06% H_2_O_2_ (Merck, Singapore) was added. 200 μL of peptide solution was then transferred to the ring cast and left at room temperature for 24 hours. Both ends of the ring were sealed with parafilm to prevent evaporation.

An ARES-G2 (TA Instruments, USA) oscillatory rheometer was used to quantify the stiffness of the gels. The elastic modulus (G′) was recorded at a strain, γ, of 0.1% and at various angular frequencies, ω (0–100 rad/s).

### Circular dichroism (CD)

LK_6_C (+/− CRGD) hydrogels were first formed overnight at 10 mg/mL in the presence or absence of 0.06% H_2_O_2_. The appropriate diluent was then added to achieve the intended concentration for CD measurements. A quartz cuvette (5 mm pathlength) and a model 410 spectrometer (Aviv Biomedical, Inc., NJ, USA) were used.

### Field-emission scanning electron microscopy (FESEM)

LK_6_C + CRGD hydrogels were freeze-dried and surface coated with platinum before its nanofibrous network was observed with a Jeol JSM-7400F (Tokyo, Japan) electron microscope.

### Direct Magnusson-Kligman maximization test

This test was administered by a contract research organization (CRO), Toxikon Corporation (MA, USA). It served to evaluate the allergenic or sensitizing potential of LK_6_C when applied directly to the skins of Hartley guinea pigs. LK_6_C was used here at a sub-gelation concentration in water (5.6 mg/mL) to facilitate handling and application. This study was performed according to ISO 10993-10 guidelines and conformed to FDA code of federal regulations title 21, part 58 – good laboratory practice for nonclinical studies. Briefly, skin test areas were prepared and subjected to a regime consisting of an induction phase by intradermal injection; a topical application phase; followed by a challenge phase. Animals were randomized into the test, negative control and positive control groups, as mentioned in the main text. At the end of treatment, the test areas were monitored for three further days and graded according to the Magnusson and Kligman scale as described in Table 2. The detailed protocol and report of this study are available upon request.

### Rate of water lost through Tegaderm-sealed containers

200 μL of water or LK_6_C + CRGD gel was filled into a container and Tegaderm was used to seal the opening. The bottom of the containers was left in contact with a heating block maintained at 37 °C, while the top was exposed to ambient conditions to simulate the wound/air interface. The rate of water lost was estimated by recording regular weight readings of the containers. All containers were allowed to dehydrate completely so as to obtain their dry weights for normalization.

### Full-thickness injury animal model and immunohistochemistry (IHC)

LK_6_C (10 mg/mL) +CRGD (1 mg/mL) hydrogels were prepared for the *in vivo* wound healing study as follows: 200 μL of the peptide solution containing 0.06% H_2_O_2_ was filled into each well of a sterile Nunc Lab-Tek II Chamber Slide System (Thermo Scientific, Sinagpore) and left to gel overnight. A dialysis-inspired strategy, previously reported and quantified^27^, was used to remove excess H_2_O_2_. For example, 500 μL of sterile water would first be layered on top of the gel. The water was then replaced by another 2 × 500 μL of water, followed by 4 × 500 μL of Dulbecco’s Modified Eagle’s Medium (DMEM) without phenol red (Invitrogen, Singapore) and with 10% FBS (Invitrogen), referred to as completed medium. When desired, medium alone (DMEM without FBS) or water was used instead of completed medium. The gel was left overnight after the last change of medium. Before application, all medium was aspirated and the chambers were detached from the slides to leave behind sterile and ready-to-use gel blocks.

The animal protocol was approved by Institutional Animal Care and Use Committee, Biological Resource Centre, Singapore. Briefly, test areas were prepared by shaving hair off the backs of 6–9 weeks old female C.B-17 SCID mice anesthetized by isoflurane inhalation (see Fig. 4a). A template was used to mark out the 1 × 1 cm square wound boundary before the epidermis and dermis were surgically removed. Next, either the DuoDerm Hydroactive gel (ConvaTec, USA) positive control or a block of the test material (LK_6_C + CRGD gel as described above) was applied to the wound. The wound was then covered up with Tegaderm (3 M, Singapore). Animals in the negative control group received no gel and just had their wounds covered up by Tegaderm. All animals received subcutaneous injections of Enrofloxacin (10 mg/kg) daily for three days post surgery. Meloxicam (for pain relieve) was laced into drinking water and made available to the mice. However, since the drug left a bitter taste, regular drinking water was provided concurrently to prevent dehydration. Mice were sacrificed on day 3, 7 and 14 by CO_2_ poisoning and thin cross-sections of the wounds at maximum diameter were prepared on glass slides. The following stainings were performed: hematoxylin and eosin (H&E), Masson’s trichrome and keratin 14 (K14) protein (monoclonal antibody LL001^37^, produced in-house). Standard IHC techniques were used and images of the stained tissues were captured with an Olympus IX-83 (Tokyo, Japan) inverted microscope.

### Scoring of tissue and statistical analysis

To discriminate between treatment groups, a scoring system based mainly on the extent of epithelial recovery was devised, as described in Fig. 7a,b. Three observers independently scored each tissue and the average score was reported. One way ANOVA followed by post-hoc Holm-Bonferroni analysis was then performed on the mean scores to determine if the completed medium-infused peptide hydrogel group is statistically different from other treatment groups. A p < 0.05 (denoted by *) was accepted to be statistically significant.

## Additional Information

**How to cite this article**: Seow, W. Y. *et al.* Transparent crosslinked ultrashort peptide hydrogel dressing with high shape-fidelity accelerates healing of full-thickness excision wounds. *Sci. Rep.* **6**, 32670; doi: 10.1038/srep32670 (2016).

## Supplementary Material

Supplementary Information

## Figures and Tables

**Table 1:** A library of peptide sequences and physical properties.

Full peptide sequence^a^	Peptide code for this study	Lowest mg/mL tested to gel^b^	Preferred working conc^c^ [mg/mL]	G′ of gel before ox^d^ [kPa]	G′ of gel after ox^e^ [kPa]	Remarks
LIVAGKC (A)	LK_6_C	8	10	3.2 ± 0.4	20.3 ± 2.0	Clear
LIVAGKC (T)	LK_6_C (T)	10	12	1.7 ± 0.2	18.0 ± 5.7	Clear
LIVAGDC	LD_6_C	1	6	Not fully soluble; inhomogeneous gel		
LIVAGSC	LS_6_C	2	4	Not fully soluble; inhomogeneous gel		
LIVAGC	LC_6_	Not soluble at 2 mg/mL; no gel formed within 24 hours				
ILVAGKC	IK_6_C	13	15	1.2 ± 0.6	1.1 ± 0.3	Translucent
IVKC	IVKC	20	25	3.6 ± 0.5	314.2 ± 114.5	Clear
IVDC	IVDC	3	7	5.4 ± 2.2	5.1 ± 3.3	Translucent
IVSC	IVSC	Not soluble at 2 mg/mL; no gel formed within 24 hours				

^a^The full peptide sequence given in single-letter amino acid code. All peptides were acetylated at their N-termini and prepared as trifluoroacetic acid salts except for LK_6_C, which was prepared either as trifluoroacetic acid (T) or acetic acid (A) salts.

^b^Peptide powder was vortexed directly in water and a gel was visually judged to be formed if it could support its weight in an overturned vial in < 24 hours.

^c^Recommended minimum working concentration of gel to facilitate handling. Elastic modulus, G′, of gel formed.

^d^In water only (PBS for IVKC).

^e^In water (PBS for IVKC) containing 0.06% H_2_O_2_ and maintained at room temperature for 24 hours. All gels were formed at their preferred working concentration. Values shown were obtained at 0.1% strain, an angular frequency, ω, of 100 rad/s and reported as average ± s.d. of triplicates at least.

**Table 2:** The direct Magnusson-Kligman maximization test was conducted to elucidate the allergenic potential of LK_6_C.

Experimental group	Guinea pig no. (sex)	Skin score^a^	Body weight gain [g]^b^	Signs of toxicity
LK_6_C test group	1 (M)	0	272.9	—
	2 (M)	0	195.3	—
	3 (M)	0	210.2	—
	4 (M)	0	161.1	—
	5 (M)	0	231.3	—
	6 (F)	0	181.4	—
	7 (F)	0	147.3	—
	8 (F)	0	126.9	—
	9 (F)	0	172.7	—
	10 (F)	0	185.6	—
Negative control (PBS)	11 (M)	0	198.5	—
	12 (M)	0	252.3	—
	13 (M)	0	209.4	—
	14 (F)	0	283.0	—
	15 (F)	0	237.3	—
Positive control (Dinitrochloro—benzene)	16 (M)	1	241.2	—
	17 (M)	1	162.7	—
	18 (F)	1	177.5	—
	19 (F)	1	165.1	—
	20 (F)	1	183.2	—

All test materials were applied directly to the skins of guinea pigs and test areas were graded according to the Magnusson and Kligman scale. All animals gained weight at the point of sacrifice and only the positive control group provoked an allergic reaction. LK_6_C failed to elicit any sensitivity in all animals.

^a^Test area of skin was scored on the day of sacrifice according to the Magnusson and Kligman scale: 0 = no visible change; 1 = discrete or patch erythema; 2 = moderate and confluent erythema; 3 = intense erythema and swelling. Any score >0 corresponds to a positive response.

^b^Body weight gain of the animal on the day of sacrifice relative to the start of experiment.
