# Supplementary material for: Transparent crosslinked ultrashort peptide hydrogel dressing with high shape-fidelity accelerates healing of full-thickness excision wounds
Source: Sci Rep. 2016 Sep 7;6:32670. doi: 10.1038/srep32670 (PMC5013444; doi:10.1038/srep32670)
Supplement: Supplementary Information [file srep32670-s1.doc]

**Supplementary Information**

Transparent crosslinked ultrashort peptide hydrogel dressing with high shape-fidelity accelerates healing of full-thickness excision wounds

Wei Yang Seow, Giorgiana Salgado, E. Birgitte Lane and Charlotte A. E. Hauser

Wavelength (nm)

[θ]×10^3^ (deg cm^2^ dmol^-1^)

**b**)

LK_6_C fixed at 1 mg/mL

Water

H_2_O_2_

CRGD + H_2_O_2_

Wavelength (nm)

[θ]×10^3^ (deg cm^2^ dmol^-1^)

**a**)

LK_6_C fixed at 10 mg/mL

H_2_O_2_

**Figure S1**. CD studies performed with LK_6_C. a) LK_6_C gels were formed at 10 mg/mL in the absence or presence of H_2_O_2_. Both adopted predominantly β-turn conformations, as evidenced by the characteristic minima at ~220-225 nm. b) Based on the depth of the characteristic β-turn peak at ~220 nm, LK_6_C exhibited the most pronounced β-turn character after being crosslinked by H_2_O_2_. In the presence of CRGD or without crosslinking (water), its β-turn character reduced.


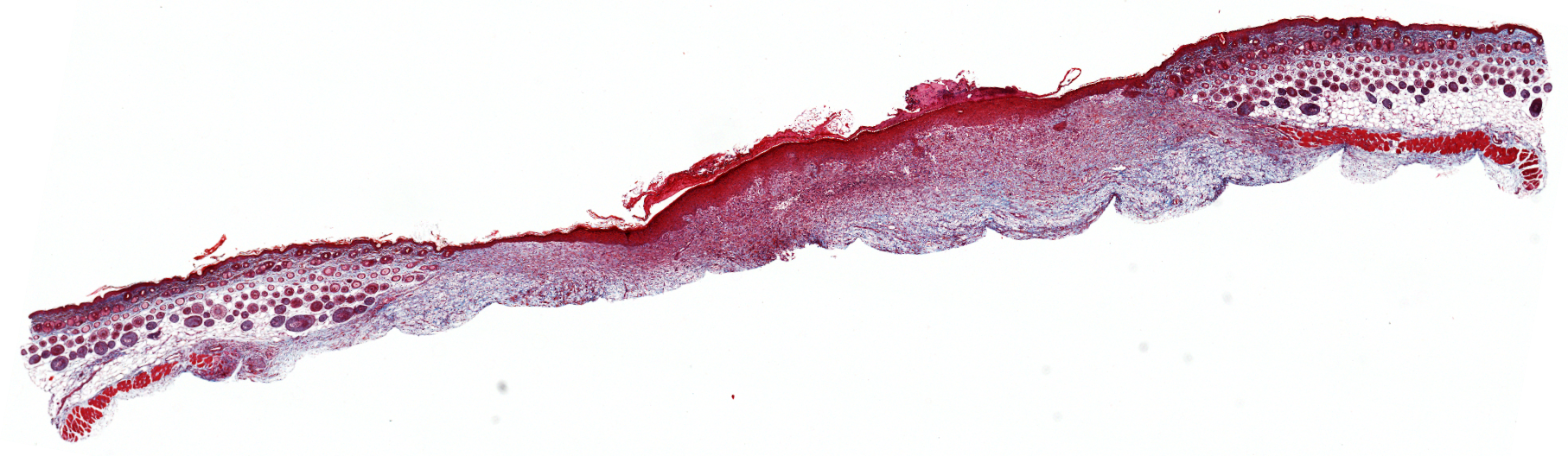


**c**) DuoDerm® control

Day 14


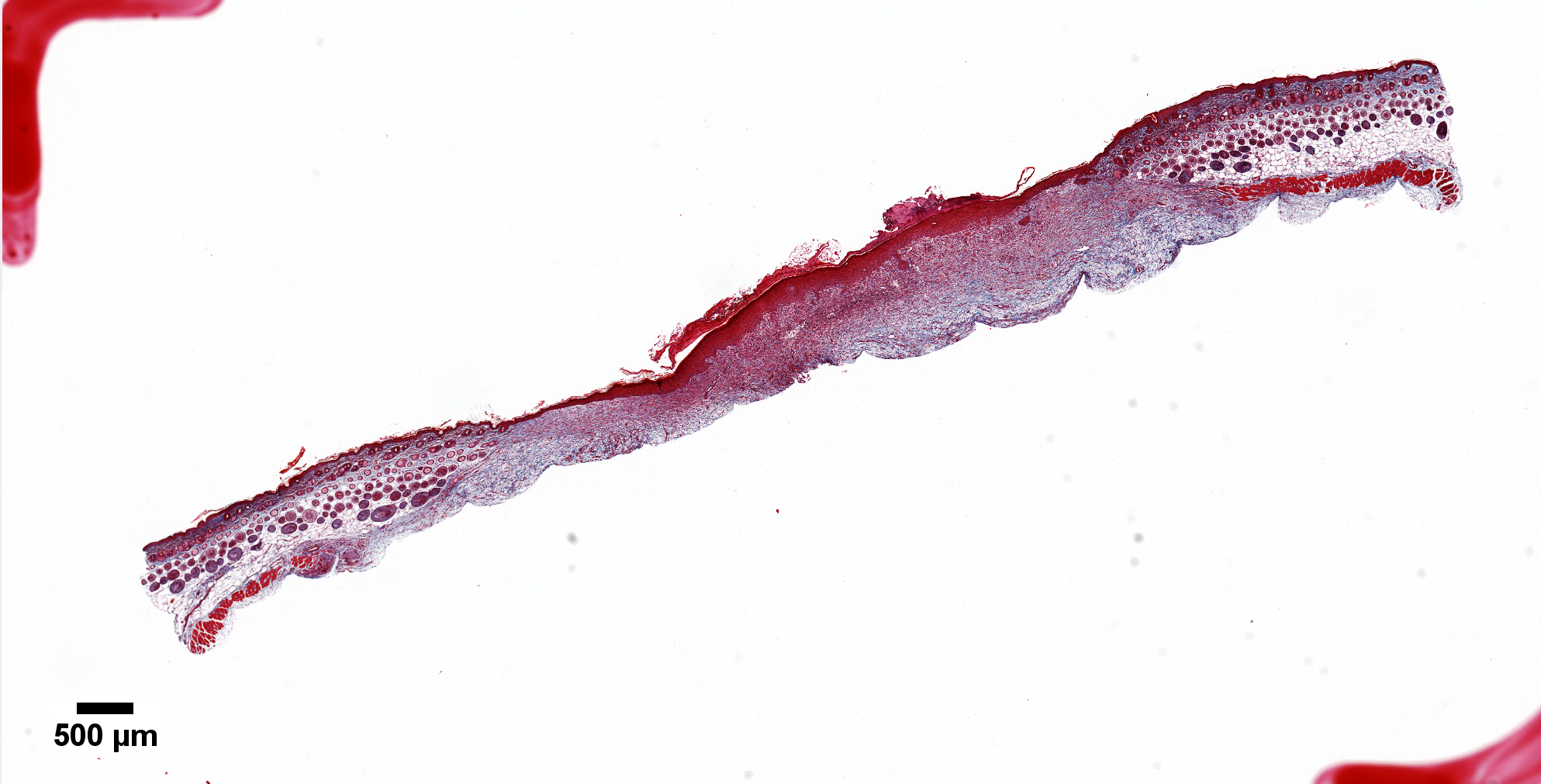

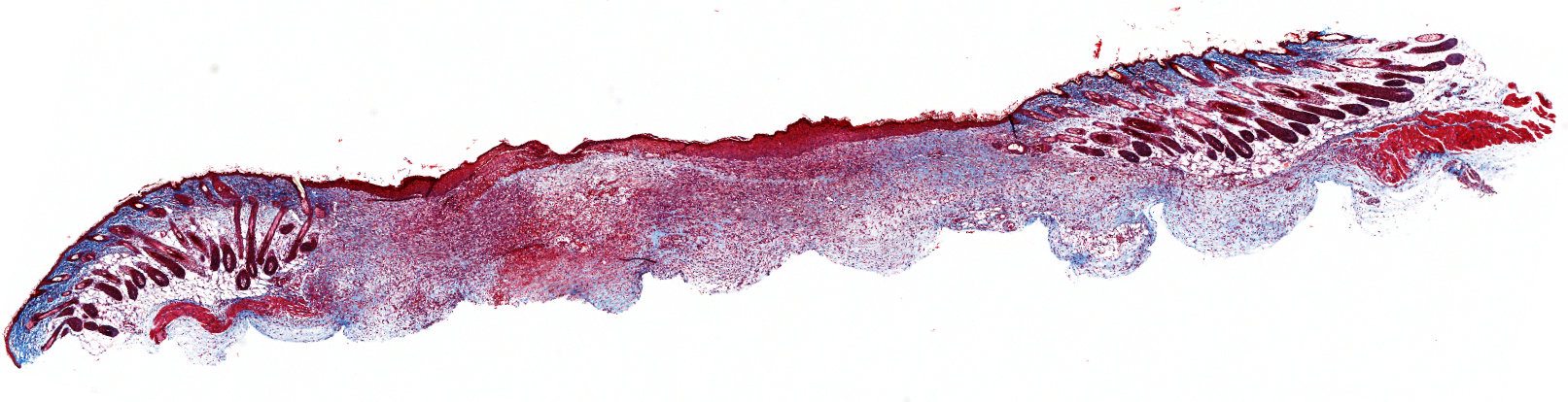


**b**) No treatment control

Day 14


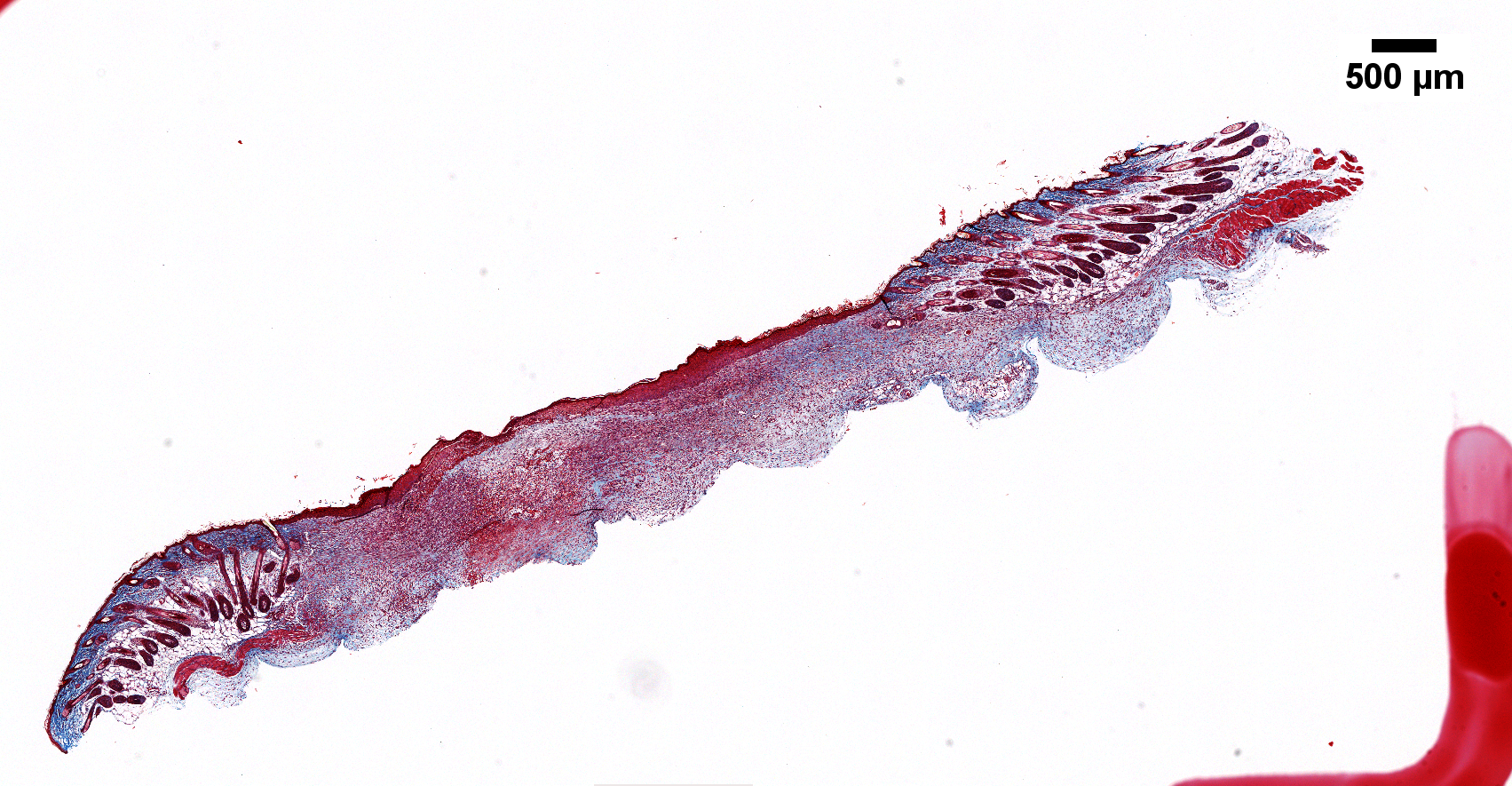

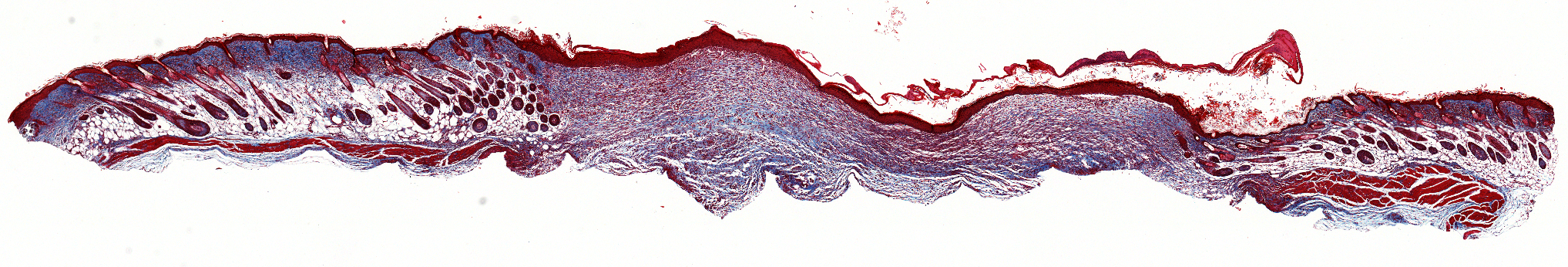


**a**) Gel + completed media

Day 14


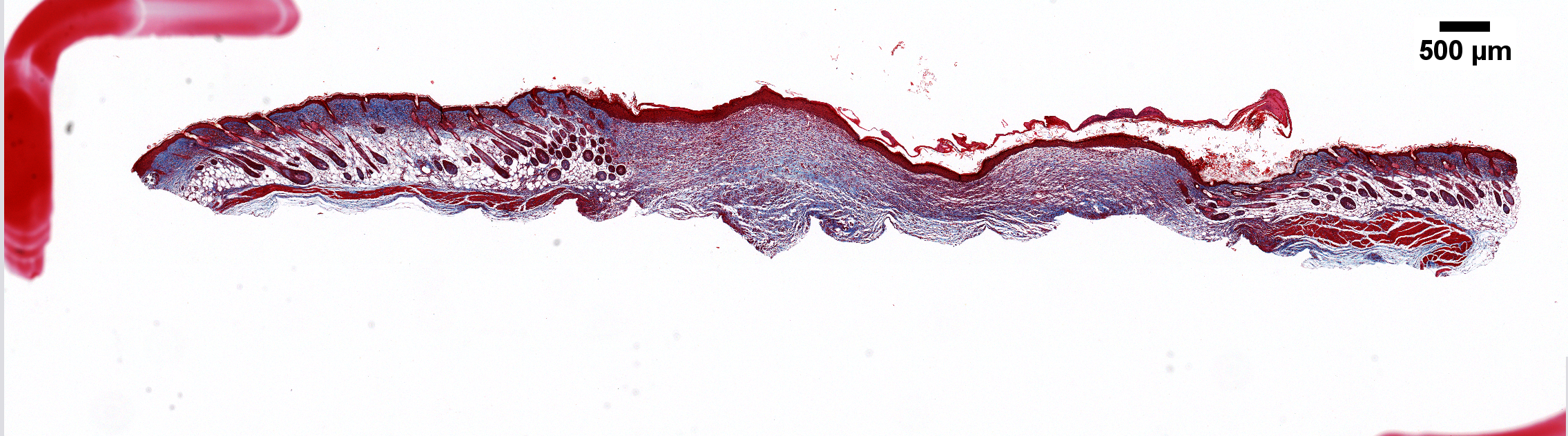


**Figure S2**. Representative paraffin-embedded cross-sections of wounds treated with a) completed medium-infused peptide hydrogel, b) no treatment and c) DuoDerm® after Masson’s trichrome staining on day 14. Collagen is stained blue. Collagen deposition was denser near the wound edge and in the deeper tissue layers, indicative of the inwards and upwards progression of wound healing. There was no obvious difference between the treatment groups in terms of density and location of collagen deposition.
